# Dual Anticoagulant/Antiplatelet Activity of Polyphenolic Grape Seeds Extract

**DOI:** 10.3390/nu11010093

**Published:** 2019-01-05

**Authors:** Michal Bijak, Agnieszka Sut, Anna Kosiorek, Joanna Saluk-Bijak, Jacek Golanski

**Affiliations:** 1 Department of General Biochemistry, Faculty of Biology and Environmental Protection, University of Lodz, Pomorska 141/143, 90-236 Lodz, Poland; joanna.saluk@biol.uni.lodz.pl; 2 Department of Haemostasis and Haemostatic Disorders, Medical University of Lodz, Poland, Mazowiecka 6/8, 92-215 Lodz, Poland; agnieszka.sut@office365.umed.pl (A.S.); anna.kosiorek@umed.lodz.pl (A.K.); jacek.golanski@umed.lodz.pl (J.G.)

**Keywords:** grape seeds, anticoagulants, polyphenols, platelet activity modulation

## Abstract

Because of the side-effects of commonly used anti-platelet and anticoagulant drugs, investigations into plant substances with similar activities are very common. Based on our own studies in recent years, we estimate that it is possible to use natural compounds to both inhibit coagulation pathway enzymes and to reduce blood platelets’ activation. As such, in our current study we wanted to verify the anti-platelet and anticoagulant properties of grape seed extract (GSE) using in vitro models. During our analysis, the following parameters were analyzed: Coagulation times, thromboelastometry assays (coagulation time, clot formation time and maximum clot firmness), aggregation of platelets and phosphorylation of vasodilator-stimulated phosphoprotein (VASP). Adenosine diphosphate (ADP)-induced aggregation was lower in GSE 7.5 µg/mL as well as in GSE 15.0 µg/mL. A similar dependence was observed in VASP assays for GSE 7.5 µg/mL and GSE 15 µg/mL. The effect on plasma coagulation tests was distinct only with GSE 15 µg/mL. All of the thromboelastometry variables were statistically significant with 15.0 µg/mL GSE concentration. Our results show, for the first time, the multi-potential effect of grape seed extract on coagulation systems, and clearly suggest that grape seed extract could be considered a promising nutraceutical in the prevention of cardiovascular thrombotic events caused by different mechanisms.

## 1. Introduction

Antiplatelet and anticoagulant drugs have been the major players in their clinical setting [1,2]. Although their efficacy is well established, their deleterious, life-threatening side-effects have also been well documented [3,4] which has prompted investigations into natural alternatives. An exploratory trial to identify new anticoagulants and anti-platelets sourced from medicinal plants is worthwhile. Plants are a good source for isolating natural compounds without unfavorable side effects, capable of inhibiting coagulation pathway enzymes and reducing blood platelet activation [5,6,7]. Epidemiological studies have provided evidence that diets rich in polyphenols, such as flavonoids, protect against vascular dysfunction, promote vascular health and reduce the risk of cardiovascular and cerebrovascular diseases [8,9,10]. Polyphenol-rich diets have also been shown to have a positive effect on the vascular system, mainly through their effects on platelets and endothelial functions [11,12].

One of the most promising sources of bio-active substances that can be used in the prevention of primary and secondary thrombotic disorders is grape seeds. Grapes—the berries of *Vitis vinifera*—have been well-known and popularly consumed since ancient times. The nutritional and medicinal values of these fruits have been confirmed by both traditional medicine and a growing number of scientific reports [13,14,15,16]. Grape seeds, a by-product of the wine and juice industries, are also a source of numerous substances having a positive effect on human health. These seeds contain about 5–8% polyphenols (depending on the variety), including the flavan-3-ol monomers: catechin, epicatechin, gallocatechin, epigallocatechin and epicatechin 3-*O*-gallate, as well as procyanidin dimers, trimers, and highly polymerized procyanidins [17,18]. Polyphenolic compounds from grape seeds have been shown to display favorable health effects, such as antioxidative [19], cardioprotective [20], immunomodulatory and antitumor activity [21].

In Jankun et al. and Correi-da-Silva et al.’s investigations estimating polyphenol anticoagulant activity, thromboelastometry was used. In the former study, inactivation of PAI-1 by tea polyphenols was presented as ineffective [22]. In contrast, the latter study used thromboelastography to show that polysulfated flavonoids featured high blood anticoagulation activity. To sum up, these compounds are potentially safe, efficient agents for anticoagulant therapy [23].

There is observational evidence that two tools—the multiplate analyzer (using multiple electrode aggregometry (MEA)), and rotational thromboelastometry (ROTEM)—could be superior to routine testing for monitoring of bleeding in surgical patients who use herbal medicines [24].

Our previous in vitro studies show that grape seed extract (GSE) modulates the coagulation process in human plasma [25]. Grape seed extract’s action on human plasma resulted in prolongation of blood plasma clotting time (APTT, PT, TT), and a reduction of thrombin-induced plasma polymerization. Additionally, this extract strongly inhibits the amidolytic and proteolytic activity of the main human coagulation enzyme, thrombin [26].

Given this, the aim of the present study was to determine, in vitro, both the anticoagulant and anti-platelet activity of grape seed extract in different experimental models.

## 2. Materials and Methods

### 2.1. Reagents

Coagulation reagents were obtained from Siemens Healthcare Diagnostics (Erlangen, Germany). Thromboelastometry reagents were purchased from Instrumentation Laboratory (Bedford, MA, USA). Reagents for blood platelet aggregation were obtained from Roche Diagnostics Gmbh, (Munich, Germany). A cytometric kit especially designed for vasodilator-stimulated phosphoprotein (VASP) assay (PLTVASP/P2Y12) was purchased from BioCytex (Marseille, France). Ultra-purity water was purchased in the laboratory using a Simplicity™ Water Purification System—Millipore (Burlington, VT, USA). All other chemicals were reagent-grade products purchased from POCh—Gliwice (Gliwice, Poland).

### 2.2. Grape Seed Extract (GSE)

The commercial extract of grape seeds (*V. vinifera*), OMNIVIR^®^, was purchased from C. E. Roeper GmbH (Hamburg, Germany). The extract was initially dissolved in 50% DMSO. The stock solution of extract was prepared in 10% DMSO in a concentration of 7.5 and 15 µg of Gallic Acid Equivalent (GAE)/mL. Its chemical composition and method of identification were described previously [25].

### 2.3. Blood Donors

Blood samples were drawn from 30 healthy volunteers (15 men and 15 women, with a mean age of 24.9 ± 8.2 years), without chronic disease and untreated by drugs. The volunteers did not use supplements or have special diets (vegetarian or vegan, etc.). The donors had not taken salicylic acid or its derivatives, nor any other non-steroid anti-inflammatory drugs for at least 2 weeks. They were also non-smokers. A complete blood count, biochemical analysis, food frequency and daily eating history (based on special prepared survey) were all collected. We established that that the range daily polyphenol intake came to 1803.4 ± 468.3 mg/day.

Samples of blood (4.9 mL) were drawn from qualified volunteers using a Sarstedt (Nümbrecht, Germany) closed vacuum system, with S-Monovette^®^ Needles (0.9 mm/38 mm) in S-Monovette^®^ tubes. The tube contained 25 μg/mL hirudin anticoagulant (Refludan^®^, Schering AG, Berlin, Germany). 

All subjects gave their informed consent for inclusion before they participated in the study. The study was performed in accordance with the guidelines of the Helsinki Declaration for Human Research, and was approved by the Committee on the Ethics of Research in Human Experimentation at the Medical University of Łódż (with Resolution No. RNN/13/07/KB).

### 2.4. Study Protocol

The samples of whole blood or plasma (obtained by centrifugation of the blood) were incubated with grape seed extract for 15 min at 37 °C before further analyses. The final concentrations of the extract were calibrated with phenolic compounds, and were 7.5 or 15 µg GAE/mL (gallic acid equivalent). The preparations were dissolved in DMSO, with the final concentrations in all studied samples and the control being 0.17%.

### 2.5. Coagulation Assays

The human plasma coagulation assays were performed using the following kits and equipment: prothrombin time (PT) and activated partial thromboplastin time (APTT) assays were performed according to the diagnostic kit’s manufacturer’s instructions. For the PT assay, a Thromborel^®^ S test was used, while the APTT test was conducted with a Pathromtin^®^ SL test. Coagulation assays were performed with a Siemens Fibrintimer II optical coagulometer (Erlangen, Germany).

### 2.6. Thromboelastometry

The thromboelastometry properties of clots were assessed using ROTEM^®^ delta analysis by Instrumentation Laboratory (USA). The blood samples were used for ex-TEM (extrinsically-activated test with tissue factor), fib-TEM (fibrin-based extrinsically activated test with tissue factor), and platelet inhibitor cytochalasin D test assays. These assay kits were obtained from Pentapharm GmbH, (Munich, Germany). Data were collected according to the manufacturer’s instructions, and the following clotting parameters registered: coagulation time (CT, s), clot formation time (CFT, s), and maximum clot firmness (MCF, mm).

### 2.7. Measurement of Human Platelet Reactivity

Whole blood impedance aggregometry was carried out using a five-channel Roche Diagnostics Multiplate^®^ analyzer (Munich, Germany), in accordance with the manufacturer’s guidelines. In each whole blood sample, platelet aggregation was monitored for up to 15 min following the addition of ADP (6.4 mmol/L) [27].

### 2.8. Estimation of Platelet VASP Phosphorylation

The response to ADP with phosphorylation of focal adhesion VASP in platelets, was also investigated using a flow cytometrical VASP assay (PLTVASP/P2Y12) from BioCytex (Marseille, France), according to the manufacturer’s instructions and as described by Rywaniak et al. [28]. The analysis was made using a BD Bioscience FACS Canto II flow cytometer (San Jose, CA, USA), with BD FACS Diva Software v. 6.1 (BD Bioscience, San Jose, CA, USA). Platelet response was expressed as platelet reactivity index (PRI).

### 2.9. Statistical Analysis

Medians and interquartile ranges (Me, IQ) are given for all parameters showing departures from normality (according to a Shapiro-Wilk W test), and mean and standard deviation for parameters showing normal distribution. The results were analyzed using a non-parametric Wilcoxon signed-rank test, a Mann-Whitney U test, a Friedman test and a parametric t-student test.

## 3. Results

### 3.1. The Impact of GSE on Platelet Reactivity

**Figure 1:**
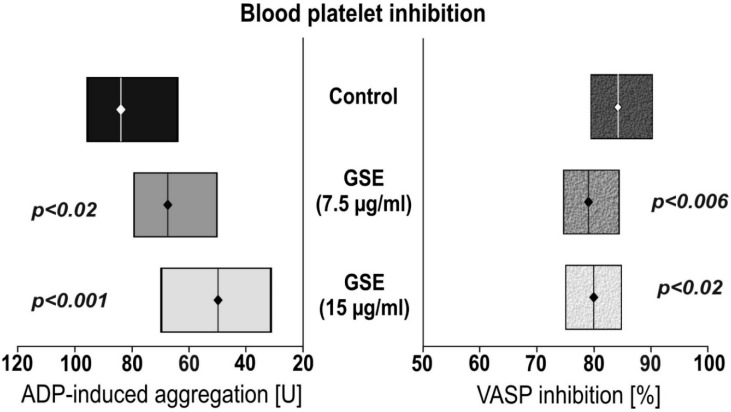
The effect of grape seed extract (GSE) on adenosine diphosphate (ADP)—induced aggregation and VASP inhibition. Aggregation was made using multiple electrode aggregometry (MEA). Vasodilator-stimulated phosphorylation (VASP) inhibition was conducted using flow cytometry. Data are presented as a median (interquartile range: LQ-UQ). The significance of the differences was analyzed using a Mann-Whitney U test.

To examine the influence of GSE on platelet responsiveness to ADP, VASP assays were performed. The platelets’ reactivity was monitored in whole-blood samples (Figure 1). We observed that incubation of whole blood with GSE decreased ADP-induced aggregation in a 7.5 µg/mL GSE concentration of 66.6 U, and 15.0 µg/mL in a 50.5 U GSE concentration. This is compared to the control of 82.7 U GSE concentration. Statistical significance was *p* < 0.012 and *p* < 0.0001 respectively. Incubation with GSE also lowered the calculated platelet reactivity index, based on VASP phosphorylation: 80.3% for 7.5 µg/mL GSE (*p* < 0.006), and 80.8% for 15.0 µg/mL GSE (*p* < 0.03) compared to the control of 85.0%.

### 3.2. The Impact of GSE on Plasma Coagulation Tests

**Figure 2:**
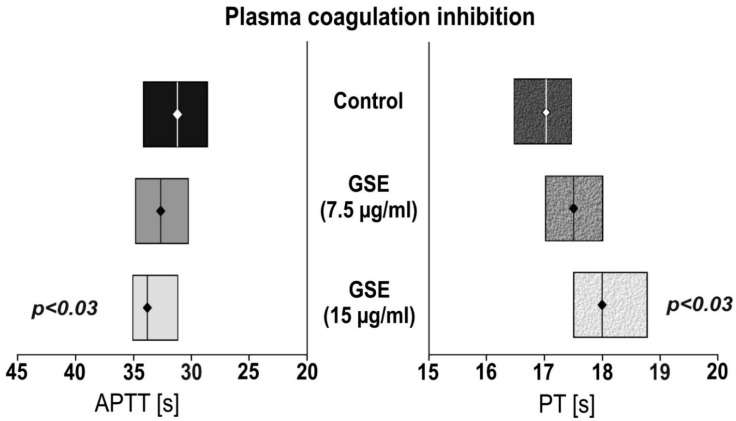
The effect of GSE on plasma coagulation tests. Activated partial thromboplastin time (APTT); Prothrombin time (PT). Data are presented as a median (interquartile range: LQ-UQ). The significance of the differences was analyzed using a Mann-Whitney U test.

The anticoagulant activity of the tested compounds was evaluated using activated partial thromboplastin time and prothrombin time assays (the results are presented in Figure 2). We observed that incubation with GSE (15µg/mL) showed statistically significant (*p* < 0.03) prolonged APTT of 33.6 s compared to the control of 31.8 s, as well as PT (*p* < 0.03) of 18.0 s versus 17.0 s. No effect was observed for the 7.5 µg/mL GSE concentration.

### 3.3. The Impact of GSE on Thromboelastometry Parameters

All of the thromboelastometry variables (CT, CFT, MCF, Amplitude-A) were significantly different after incubation with a 15.0 µg/mL GSE concentration, except for CT, which used 7.5 µg/mL GSE. The impact of GSE on thromboelastometry variables is described in Table 1.

## 4. Discussion

All around the world, continuous research is being conducted into phytochemicals with anticoagulant properties. These are considered to be therapeutically better, have more effective anticoagulant or anti-platelet agents, multiple targets, and without so many side-effects [25,29,30,31,32,33]. A review of the literature indicates that many herbals reduce clotting via inhibition of coagulation factors. Various models exist to screen for different activity; popular experiments include effects on prothrombin time, activated partial thromboplastin time, and thrombin time both in vitro and ex vivo, while bleeding time and protection against thromboembolism-induced death are monitored in vivo. Since in vitro activity does not always translate to in vivo activity, continued research in this area is essential [34,35].

In our previous studies we demonstrated that GSE (as used in the current study) presents multiple positive actions on the human haemostasis system. We demonstrated that GSE inhibits human plasma coagulation, and causes statistically significant prolonged APTT, prothrombin PT, and TT [25]. The mechanism of this phenomenon is related to the inhibition of thrombin activity. The GSE inhibited the proteolytic activity of thrombin, observed as inhibition of thrombin-induced fibrinogen polymerization, stabilized fibrin formation and platelet aggregation [36]. Additionally, OMNIVIR^®^ has strong antioxidant properties and reduces the formation of 3-nitrotyrosine carbonyl groups, and diminishes the oxidation of thiol groups in human plasma proteins [37].

In our current study we performed multistep analysis of GSE on all elements of human haemostasis. We tested both the cellular and plasma components of the blood coagulation system. We demonstrated that GSE in concentrations of 15 µg/mL is able to prolong coagulation time of APTT and PT (Figure 2), as well as inhibit blood platelet response to one of the most prothrombotic agonists, ADP (Figure 1).

Platelet activation by ADP is mediated by two G protein-coupled receptors: P2Y1 and P2Y12. The P2Y1 receptor interacts with Gq, whereas P2Y12 is coupled to the Gi-type of G proteins. Previous studies with receptor agonists have suggested that activation of both P2Y receptors is required for a full response of platelets to ADP. Generally, the P2Y1 pathway initiates platelet activation and mediates weak responses to ADP, whereas the P2Y12 receptor plays a role in strongly amplifying the platelets activation process and is essential for a full aggregation response to ADP and the stabilization of aggregates [38]. VASP is an intracellular platelet protein that is non-phosphorylated in its basal state. VASP phosphorylation is regulated by the cyclic Adenosine Monophosphate (cAMP) cascade, whereas it is inhibited by ADP through the P2Y12 receptors’ activation. Under test conditions, VASP phosphorylation correlates with P2Y12 receptor inhibition, whereas its non-phosphorylation state correlates with the active form of the P2Y12 receptor [39]. In our investigations we were focused on a platelet activation pathway dependent on ADP. With the present study, we proved that anti-platelet GSE activity is dependent on the P2Y12 receptor (increased VASP phosphorylation, reduced PRI). In the work of Rywaniak et al., a phenolic acid-rich extract of *Arnica montana* (7.5 and 15 µg/mL) significantly reduced the ADP-induced aggregation in both whole blood and PRP, and decreased the platelet reactivity index (PRI; VASP phosphorylation) in whole blood [28], which confirms our own results (Figure 1).

For the last few years, flavonoids—especially flavones, flavonols and flavanols—have been investigated as inhibitors of blood platelet aggregation. Flavonoids are capable of reducing blood platelet aggregation and the granule secretion induced by different stimulators. This effect is mediated by inhibition of different pathways, such as phosphoinositide 3-kinase (PI3K)/PKB (AKT), extracellular signal-regulated kinase (ERK) 1/2, p38 and c-Jun N-terminal kinase (JNK) ½ [40]. Inhibition of the PI3K/PKB pathway is most likely with GSE, because upon stimulation of the P2Y12 receptor the sub-units of the heterotrimeric Gi protein (Gα and the Gβγ dimer) are dissociated and activate different signaling pathways. The Gβγ dimers then activate the PI3K/PKB pathway, which results in activation of the αIIbβ3 integrin (fibrinogen receptor), and subsequent aggregation [41,42].

The last parameter established in this study was thromboelastometry. This is a global assessment of haemostatic function for investigation of the interaction of platelets with the coagulation cascade, from the time of initial fibrin formation through to platelet aggregation, clot strengthening, fibrin cross linkage, and on to eventual clot lysis. In our study, we showed that GSE is able to reduce all thromboelastometry parameters (Table 1), which confirms its strong multiple anticoagulant properties.

The bioavailability of polyphenols is an important element in the evaluation of their biological properties in vivo. Both concentrations tested in this study have been chosen based on a previous study where these concentrations showed non-cytotoxic effect [43]. The concentrations of flavonoids (1.5–6 µg/mL) present in the 15 µg/mL of GSE used in our work which have been calculated based on our chemical characterization of this extract [25] can be achieved in plasma during supplementation with these extracts. In the case of flavanols they reached plasma concentrations ranging from 1.5 to 2 µg/mL [44,45,46].

Our results show, for the first time, that the multi-potential effect of GSE on the coagulation system clearly suggests that GSE could be considered as a promising nutraceutical in the prevention of cardiovascular thrombotic events caused by different mechanisms.

## Figures and Tables

**Table 1:** The effect of GSE on thromboelastometry variables, as measured by an EXTEM test (ROTEM^®^).

Thromboelastometry Variable	Control	GSE 7.5 µg/mL	GSE 15.0 µg/mL
Clotting Time—CT (s)	48.5 (43.3; 52.9)	69.5 * (63.8; 76.0)	77.1 * (69.3; 88.0)
Clotting Formation Time—CFT (s)	94.3 (87.4; 108.9)	103.5 (86.5; 131.8)	120.5 # (98.3; 155.9)
Amplitude—A (mm)	70.5 (58.1; 72.8)	69.5 (65.0; 72.8)	67.5 & (61.0; 70.0)
Maximum Clot Firmness—MCF (mm)	58.5 (55.6; 59.9)	58.0 (64.5; 61.0)	55.0 @ (52.0; 60.0)

Data are presented as a median (interquartile range: LQ-UQ). The significance of the differences was analyzed using a Mann-Whitney U test. * CT_control_ ≠ CT-GSE_7.5 µg/mL_ and CT-GSE_15 µg/mL_–*p* < 0.0001; CT_7.5 µg/mL_ ≠ GSE_15 µg/mL_–*p* < 0.02; # CFT_control_ ≠ CFT_15 µg/mL_–*p* < 0.003; & A_control_ ≠ A_15 µg/mL_–*p* < 0.04; @ MCF_control_ ≠ MCF_15 µg/mL_–*p* < 0.05.
